# Association between frailty and hepatic fibrosis in NAFLD among middle-aged and older adults: results from NHANES 2017–2020

**DOI:** 10.3389/fpubh.2024.1330221

**Published:** 2024-02-08

**Authors:** Falide Atabieke, Xiu-Juan Li, Ailikamu Aierken, Jian Li, Yu Zhang, Yierzhati Aizezi, Hong-Liang Gao, Zhi-Qiang Zhang

**Affiliations:** ^1^The Second Department of Gastroenterology, The First Affiliated Hospital of Xinjiang Medical University, Urumqi, Xinjiang Uygur Autonomous Region, China; ^2^Department of Pathophysiology, School of Basic Medical Sciences Xinjiang Medical University, Urumqi, Xinjiang Uygur Autonomous Region, China; ^3^Xinjiang Medical University School of Clinical Medicine, Children's Hospital of the Autonomous Region, Urumqi, Xinjiang Uygur Autonomous Region, China; ^4^Center of Critical Care Medicine, First Affiliated Hospital of Xinjiang Medical University, Urumqi, Xinjiang Uygur Autonomous Region, China

**Keywords:** frailty, hepatic fibrosis, non-alcoholic fatty liver disease, national health and nutrition examination survey, controlled attenuation parameter

## Abstract

**Background:**

Although previous studies found that frailty is prevalent in NAFLD patients with advanced liver fibrosis and cirrhosis, studies examining the relationship are spare.

**Aim:**

Our study aspires to investigate the potential correlation between the Frailty Index (FI) and hepatic fibrosis among middle-aged and older adults with NAFLD.

**Methods:**

Data from the 2017–2020.03 National Health and Nutrition Examination Survey (NHANES) were utilized for this study, with a final of 2,383 participants aged 50 years and older included. The quantification of frailty was executed employing a 49-item frailty index. The recognition of hepatic steatosis and fibrosis was accomplished through the utilization of the controlling attenuation parameter (CAP) and transient elastography (TE). The relationship between the FI and hepatic fibrosis were investigated employing univariable and multivariable-adjusted logistic regression analyses. A subgroup analysis was conducted, dividing the subjects based on gender, Body Mass Index (BMI), and the presence of hyperlipidemia.

**Results:**

The findings demonstrated a positive correlation between the FI and significant hepatic fibrosis in NAFLD, even after using multivariate logistic regression models adjusting for potential confounding factors (OR = 1.022, 95% CI, 1.004–1.041) and in tertiles (Q3vs Q1: OR = 2.004, 95% CI, 1.162–3.455). In the subgroup analysis, the correlation was more statistically significant in male (OR = 1.046, 95% CI, 1.022–1.071), under/normal weight (OR = 1.077, 95% CI, 1.009–1.150), overweight (OR = 1.040, 95% CI, 1.010–1.071), and subjects without hyperlipidemia (OR = 1.054, 95% CI, 1.012–1.097). The area under the Receiver Operating Characteristic (ROC) curve for the FI in assessing the existence of substantial fibrosis in NAFLD was 0.612 (95% CI, 0.596–0.628).

**Conclusion:**

This study demonstrated a positive correlation between significant hepatic fibrosis and frailty, particularly among males aged 50 years and older, who were non-obese and did not have hyperlipidemia with NAFLD. Additional studies are required to further validate these findings.

## Introduction

Non-alcoholic fatty liver disease (NAFLD) is generally acknowledged as the liver representation of metabolic syndrome (1), affecting a spectrum of hepatic conditions in individuals who consume little to no alcohol. The defining characteristic of NAFLD is the accumulation of excessive fat within liver cells. Recent research suggests that the emergence of NAFLD is associated with the accumulation of lipids, endoplasmic reticulum stress, oxidative stress, lipotoxicity within the liver (2). If left untreated, the condition can potentially lead to hepatic fibrosis, cirrhosis and ultimately, hepatocellular carcinoma (3). Consequently, preventing the advancement of fibrosis can serve as a crucial measure to reduce liver-related mortality. Non-invasive assessment methods are increasingly being recognized as alternatives besides of liver biopsy (4). Transient elastography (TE), delivering accurate staging of liver fibrosis in NAFLD using non-invasive methods, is a promising technique, particularly for advanced fibrosis and cirrhosis. Controlled attenuation parameter (CAP) method is routinely used to determine steatosis severity and also being studied for the grading of hepatic steatosis (5, 6).

Frailty, which was marked by age-related reduced functional reserves through multiple organ systems, is a prevalent and significant geriatric syndrome and can result in heightened susceptibility to negative health outcomes (7). Understanding the risks of frailty and associated adverse health outcomes can help to better treat this most vulnerable group of patients. Although there is no gold standard for detecting frailty, a variety of screening tools for frailty have been developed and used in risk assessment and epidemiologic studies (8). The frailty index (FI) is calculated based on the presence or absence of multiple health-related deficits or impairments (9), such as chronic diseases, disabilities, cognitive decline, or other age-related conditions. The FI provides a numerical score or index that represents the overall frailty status of an individual, with higher scores indicating greater frailty. A total of 49 health indicators were incorporated to create the FI as a ratio of accumulated health deficits.

In the previous studies, researchers found that frailty is prevalent in NAFLD patients with advanced liver fibrosis and cirrhosis (10). In this study, we analyzed the relevance of the FI and hepatic fibrosis among middle-aged and older adults in US with NAFLD using the 2017–2020.03 National Health and Nutrition Examination Survey (NHANES) data.

## Methods

### Study design and participants

The NHANES is a nationally representative database, which delivers comprehensive data regarding nutrition and health for the common U.S. population (11). The technique and data acquisition process of NHANES have been thoroughly detailed in prior publications, and can be accessed on the official NHANES website^1^ (12). 2017–2020.03 NHANES survey cycles were selected due to the availability of specific data on assessment of hepatic fibrosis is not available in the former waves. TE, which is capable of executing with remarkable diagnostic precision, regardless of the underlying liver condition, for the identification of cirrhosis, was used to assess hepatic fibrosis in our study (13). In a study executed by Karlas et al. (6), a CAP score of ≥248 dB/m was recognized as an indicator of NAFLD, and individuals without NAFLD were not included. Based on the latest guidelines of the European Association (14), a median liver stiffness of ≥8.2 kPa was used to judge significant hepatic fibrosis (≥F2). Individuals were deemed ineligible if they could not lie on the examination table, were pregnant or uncertain about their pregnancy status during the testing period.

**Figure 1:**
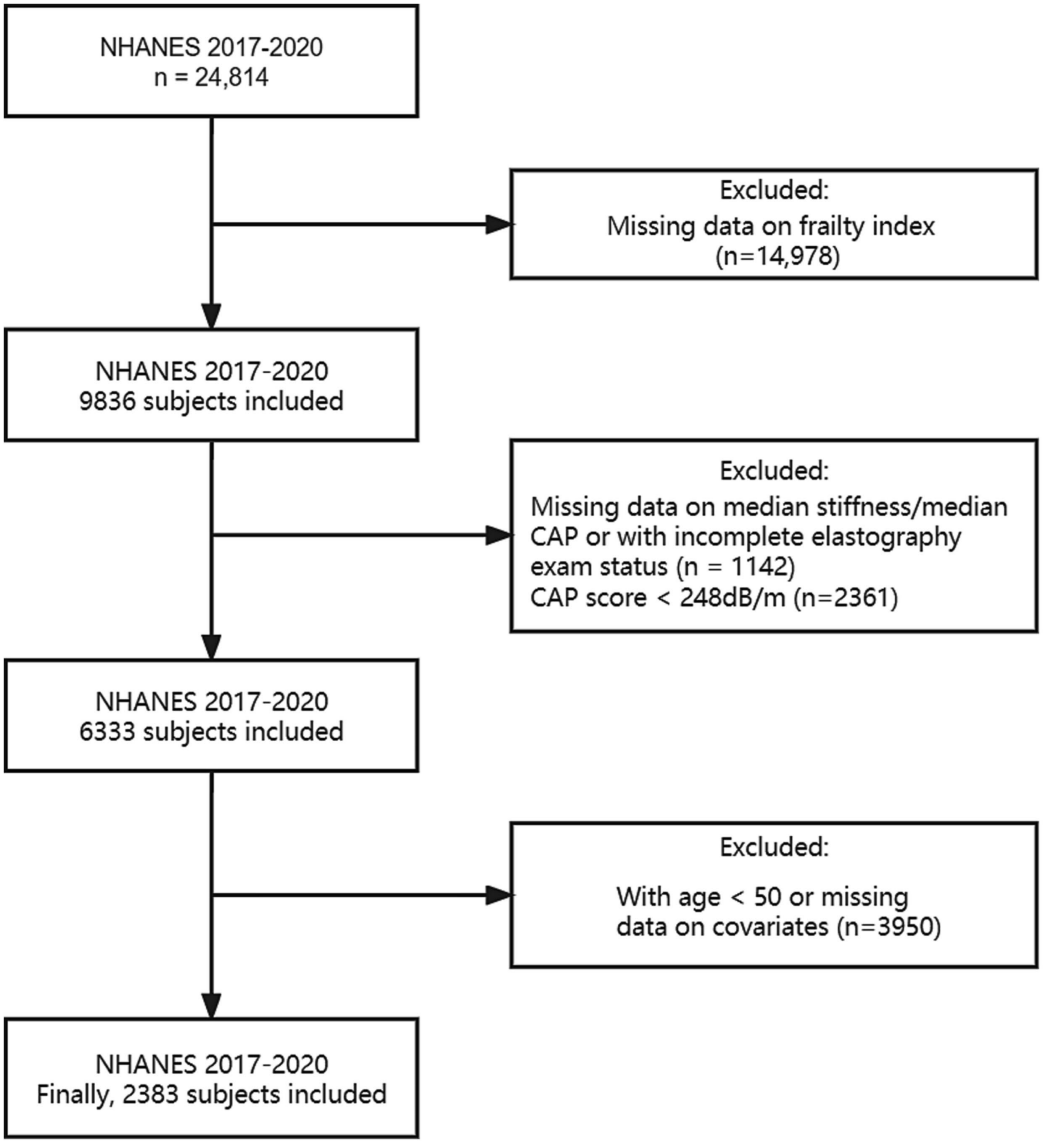
Flow chart for inclusion and exclusion.

Weighted 2017–2020.03 cycles were calculated and utilized throughout the analysis due to the Covid-19 pandemic. We included individuals aged 50 years and older who had no other potential causes of chronic liver disease such as hepatitis B, hepatitis C, liver cancer, autoimmune hepatitis, or serious alcoholism. In the NHANES cycles from 2017 to 2020.03, 24,814 individuals participated in the study, with 8,056 of them being 50 years old or older. While excluding subjects without data on assessment of hepatic fibrosis or the FI, as well as any other covariates such as age, gender, race/ethnicity, educational level, body mass index (BMI), smoking status and alcohol behavior, 2,383 remaining sample was used for analysis. Details are shown in Figure 1.

### Detection methods

The primary object of TE is offering a reliable detection method for 2 significant hepatic diseases: hepatic fibrosis, hepatic steatosis. The elastography measurements were conducted in the Mobile Examination Center (MEC) of the NHANES, using the FibroScan^®^ model 502 V2 Touch equipped with a medium (M) or extra large (XL) wand. Simultaneously, the ultrasound attenuation associated with hepatic steatosis was also assessed, and the index of hepatic steatosis was recorded from CAP. A meta-analysis included 19 biopsy control studies in more than 2,700 patients suggested that the best critical value for liver steatosis grade was 248 dB/m (95% CI 237–261) (6). Others have evaluated elastography for its accuracy in assessing hepatic steatosis and fibrosis (15).

### Frailty index

The FI is an integrative assessment tool designed to appraise the degree of vulnerability to adverse outcomes typically in the context of aging and health, which counts 49 deficits in health that covered multiple systems constructed by Hakeem FF (16). The FI computation encompassed the incorporation of symptoms, signs, disabilities and diseases in this study (17). These deficits encompassed limited activity, cognitive impairments and physical performance deficits (such as weakened grip strength, difficulty walking), co-existing medical conditions, self-assessed health status, and mood/depression issues (18). Depending on the severity of the deficit, a value between 0 and 1 was assigned. The FI value represents the ratio of deficits acquired by the participant to the sum of potential deficits. Consider a scenario where 40 potential deficits are evaluated, if an individual exhibits 10 of these deficits, his frailty index would be calculated as 10/40 = 0.25 (18). It’s important to note that the probability of an individual being categorized as frail escalates in direct proportion to the number of deficits they manifest. The variables of the FI with their corresponding values are shown in Supplementary Table S1.

### Covariate

Several factors were scrutinized as potential confounders, and were duly incorporated as adjustments within the analytical framework. Demographic data included age, sex, race/ethnicity and education level. Race/ethnicity categories were “non-Hispanic white,” “non-Hispanic black,” “Mexican American,” “other races.” Educational level included “<high school” and “≥high school.” BMI, smoking status and alcohol behavior were evaluated as health conditions and lifestyle habits.

The smoking status classification divides the population into three segments based on whether or not they have smoked 100 cigarettes: never, former, and current smokers. Former and current smokers were differentiated according to whether or not they currently smoked (19). The alcohol behavior was divided into three separate categories, each representing unique patterns of alcohol consumption. Individuals who claimed to have consumed fewer than 12 alcoholic beverages throughout their lifetime were never-drinkers. Moderate drinkers were defined as 2 or more alcoholic beverage consumption in women or 3 or more in men per day. 3 or more alcoholic beverage consumption daily in women, or 4 or more drinks per day in men, combining a minimum of 5 binge drinking episodes per month were defined as heavy drinkers (20). Participants needed to be excluded if they were heavy drinkers. BMI was calculated by dividing the individual’s measured weight in kilograms (kg) by the square of their measured height in meters (m^2^), participants were divided into three categories and their corresponding values were: under/normal weight (<25 kg/m^2^), overweight (25–29.9 kg/m^2^) or obese (>30 kg/m^2^). Hyperlipidemia (yes or no), globulin level (g/dL) and median CAP (dB/m) were also inserted in the adjustments. Hyperlipidemia was defined as triglycerides ≥150 mg/dL, total cholesterol ≥200 mg/dL, low-density lipoprotein (LDL) ≥130 mg/dL or high-density lipoprotein (HDL) ≤50 mg/dL in females and ≤ 40 mg/dL in males according to the National Cholesterol Education Program (21). All covariates are presented in Table 1.

**Table 1:** Characteristics of participants with NAFLD by significant hepatic fibrosis status in the 2017–2020 NHANES.

		Significant fibrosis		
Characteristic	Overall, (*n* = 2,383)^a^	No, (*n* = 2001)^a^	Yes, (*n* = 382)^a^	*p* value^b^
Age (years)	62.88 ± 8.63	62.92 ± 8.53	62.63 ± 9.22	0.5
Gender, *n* (%)				0.7
Male	49.57	49.80	48.22	
Female	50.43	50.20	51.78	
Race/ethnicity, *n* (%)				0.011
Non-Hispanic White	71.60	72.78	64.37	
Non-Hispanic Black	7.83	7.40	10.47	
Mexican American	5.38	4.91	8.29	
Other	15.19	14.92	16.87	
Education level, *n* (%)				0.6
High school or above	90.75	90.91	89.75	
Less than high school	9.25	9.09	10.25	
BMI group, *n* (%)				<0.001
Under/normal weight	11.76	13.05	3.92	
Overweight	31.96	34.99	13.44	
Obese	56.27	51.96	82.65	
Smoking status, *n* (%)				0.10
Former	30.00	29.09	35.59	
Never	62.50	63.00	59.43	
Now	7.50	7.91	4.98	
Alcohol behavior, *n* (%)				0.086
Mild	65.88	66.02	65.03	
Moderate	22.37	23.06	18.12	
Never	11.75	10.92	16.85	
Hyperlipidemia, *n* (%)				0.7
No	15.73	15.93	14.51	
Yes	84.27	84.07	85.49	
Globulin (g/dL)	2.97 ± 0.40	2.95 ± 0.38	3.12 ± 0.47	0.001
Median CAP (dB/m)	307.31 ± 40.87	303.46 ± 39.17	330.89 ± 43.16	<0.001
FI	17.75 ± 9.85	17.29 ± 9.83	20.60 ± 9.48	<0.001

a Mean ± SD for continuous; *n* (%) for categorical.

b Wilcoxon rank-sum test for complex survey samples; chi-squared test with Rao & Scott’s second-order correction.

## Statistical analysis

Participants involved in this study were summarized and compared by groups with or without significant hepatic fibrosis. Continuous variables are expressed as mean ± SD, and categorical variables are presented as numbers (percentage). The Wilcoxon rank-sum test was used to test continuous data and linear regression analysis (coefficients and 95% confidence intervals) was performed to see the association between hepatic fibrosis with globulin level, median CAP and the FI. Chi-square test was used to calculate the difference in categorical variables presented as numbers (percentage) by group. The independent correlation between the frailty index and significance of hepatic fibrosis was calculated using multivariate logistic regression models by calculating odds ratios (ORs) and corresponding 95% confidence intervals (CIs). In order to minimize the risk of excessive adjustment for confounding variables that may mediate the relationships between the FI and significant hepatic fibrosis, we constructed three models. No variable was adjusted in model 0. Age, sex and race were adjusted in model 1. In model 2, age, sex, race, educational level, smoking status, alcohol behavior were adjusted. In model 3, BMI, hyperlipidemia, and globulin level was further adjusted. The FI (as continuous variable) was further divided into tertiles, and the lowest tertile serves as the reference group. Additionally, subgroup analyses were conducted, stratifying the subjects by gender, BMI, and hyperlipidemia. A value of *p* < 0.05 (two-sided) indicates statistical significance. We multiplied the frailty index by 100 to yield integer values. All analyses were performed using R (version 4.3.1).

## Results

### Study participants and baseline characteristics

**Figure 2:**
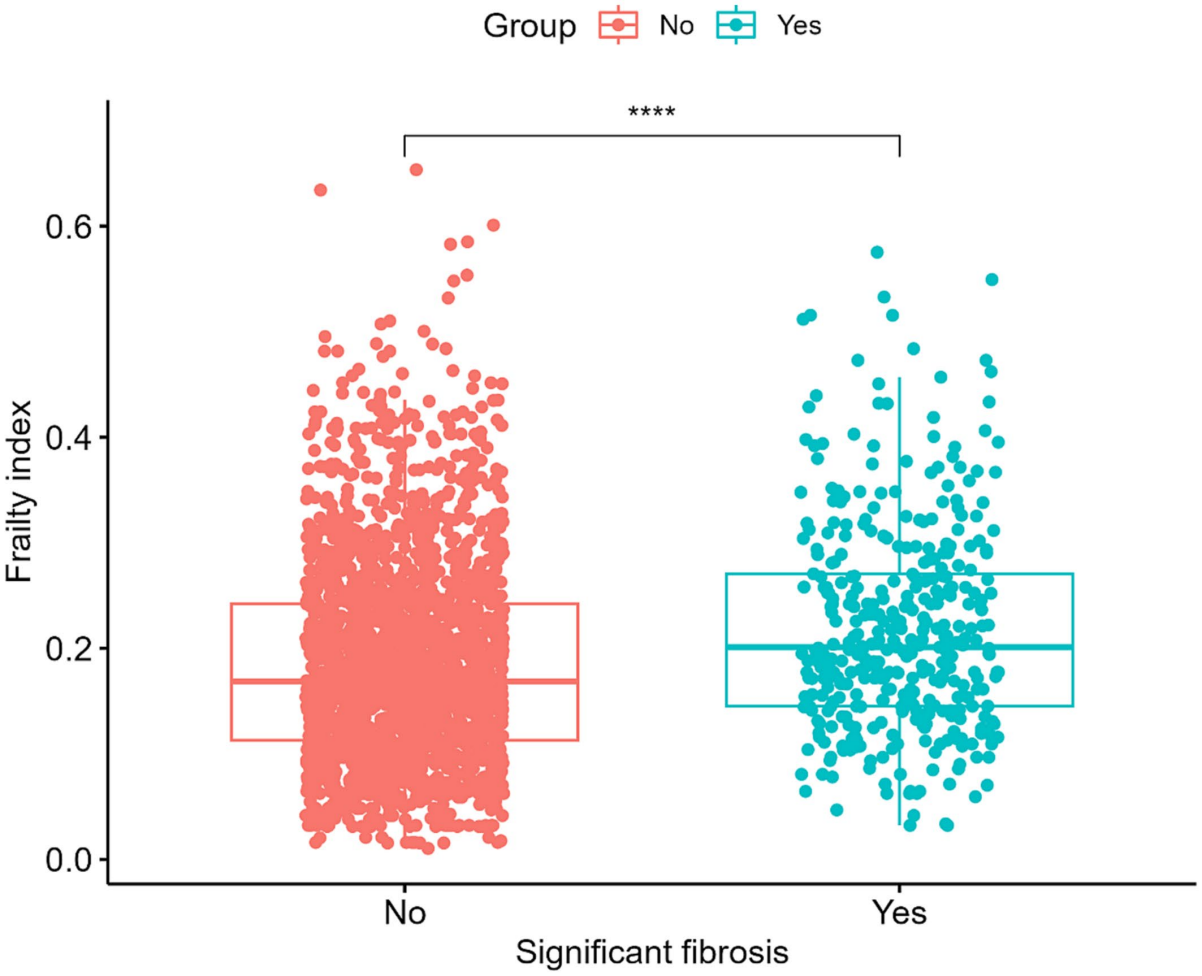
Levels of the frailty index in patients with and without significant hepatic fibrosis. ****p < 0.0001.

Our study ultimately included 2,383 participants, of which 382 participants with significant fibrosis in NAFLD. 61.69% used medium (M) wand (*n* = 1,470), while 38.31% used extra large (XL) wand (*n* = 913), 16.03% subjects with NAFLD have significant fibrosis, and the characteristics of the participants are presented in Table 1. The average age of the population was 62.88 ± 8.63, and 50.43% were female. Statistically significant differences were observed in race, BMI, globulin levels, median CAP and FI between the two groups with or without significant fibrosis (*p* < 0.05). Specifically, the group with significant fibrosis had a higher proportion of females, obese, former smokers, never had alcohol behavior, higher globulin levels and higher FI as delineated in Table 1 and Figure 2.

### Associations between the FI and significant fibrosis

A linear regression analysis was undertaken to explore the association between hepatic fibrosis and the variables in question among middle-aged and older adults, details are shown in Table 2. In the context of multivariate analysis, demographic attributes such as gender (*p* = 0.194) and educational level (*p* = 0.599) did not exhibit a significant correlation with the presence of hepatic fibrosis. However, age and races were discernibly linked with the status of hepatic fibrosis. Smoking status and BMI were associated with fibrosis (*p* < 0.001), while alcohol behavior was not (*p* = 0.135). There was a notable statistical correlation observed between the FI and hepatic fibrosis.

**Table 2:** Risk factors for significant hepatic fibrosis.

Variable	β	Standard error	95% CI	*p* value
Age	−0.025	0.011	(−0.049 to-0.002)	0.038
Sex	0.257	0.198	(−0.151 to 0.665)	0.206
Races	1.278	0.391	(0.474 to 2.083)	0.003
Education	−0.235	0.466	(−1.155 to 0.685)	0.603
Smoke	−1.232	0.246	(−1.738 to-0.726)	0.001
Alcohol	−0.415	0.291	(−1.015 to 0.185)	0.167
BMI	1.551	0.338	(0.856 to 2.247)	0.001
Hyperlipidemia	0.142	0.282	(−0.439 to 0.723)	0.62
Globulin	0.975	0.341	(0.272 to 1.678)	0.008
FI	0.564	0.139	(0.278 to 0.850)	0.001

BMI, body mass index; FI, frailty index.

Three multivariable logistic regression analysis were constructed to examine the association between the FI and significance of hepatic fibrosis. In model 3, when considering the FI as a continuous variable, a one standard deviation increase in the FI was associated with an adjusted odds ratio (OR) of 1.022 (95% CI, 1.004–1.041) for significant fibrosis. Participants in the higher two tertiles of the FI displayed a significantly elevated risk of significant fibrosis when compared to those in the lowest tertile (Q1). Moreover, a positive correlation was observed between the FI and the presence of significant fibrosis in both the second (Q2) and third (Q3) tertiles. Additionally, this correlation remained significant even after controlling for potential confounding factors in model 2 (Q3 vs. Q1: OR = 2.874, 95% CI, 1.698–4.866) and model 3 (Q3vs Q1: OR = 2.004, 95% CI, 1.162–3.455). Details are presented in Table 3.

**Table 3:** Association between the frailty index and significant hepatic fibrosis in NAFLD.

Variable	Event/total	OR (95% CI)			
		Model 0^a^	Model 1^b^	Model 2^c^	Model 3^d^
FI	382/2383	1.032 (1.018, 1.046)^***^	1.033 (1.017, 1.050)^***^	1.036 (1.019, 1.053)^***^	1.022 (1.004, 1.041)^*^
FI (tertiles)					
Q1	79/792	Ref.	Ref.	Ref.	Ref.
Q2	140/798	2.745 (1.659, 4.540)^**^	2.852 (1.690, 4.815)^**^	2.859 (1.699, 4.812)^**^	2.386 (1.425, 3.994)^**^
Q3	163/793	2.688 (1.694, 4.268)^**^	2.792 (1.684, 4.627)^**^	2.874 (1.698, 4.866)^**^	2.004 (1.162, 3.455)^*^

a Model 0 no variable was adjusted.

b Model 1 adjusted for Age, Sex, and Races.

c Model 2 adjusted for Age, Sex, Races, Education, Smoke, and Alcohol.

d Model 3 adjusted for Age, Sex, Races, Education, Smoke, Alcohol, BMI, Hyperlipidemia, and Globulin.

*p < 0.05, **p < 0.01, ***p < 0.001.

Nonetheless, upon stratification by gender, BMI, and hyperlipidemia, this association was not statistically significant among female (OR = 0.996, 95% CI, 0.970–1.022), obese (OR = 1.016, 95% CI, 0.995–1.038) and participants with hyperlipidemia (OR = 1.018, 95% CI, 0.999–1.038). The association between the FI and significant fibrosis in male has a similar result of the total population (OR = 1.046, 95% CI, 1.022–1.071) shown in Table 4.

**Table 4:** Association between the frailty index and significant hepatic fibrosis in NAFLD by gender, BMI and hyperlipidemia.

Variable	OR (95% CI)		
	Model 1^a^	Model 2^b^	Model 3^c^
Stratified by gender^d^			
Male	1.057 (1.031, 1.082)^***^	1.058 (1.031, 1.086)^***^	1.046 (1.022, 1.071)^***^
Female	1.012 (0.990, 1.033)	1.014 (0.991, 1.039)	0.996 (0.970, 1.022)
Stratified by BMI			
Under/normal weight	1.029 (0.923, 1.148)	1.072 (1.009, 1.140)^*^	1.077 (1.009, 1.150)^*^
Overweight	1.046 (1.019, 1.073)^***^	1.042 (1.012, 1.072)^**^	1.040 (1.010, 1.071)^**^
Obese	1.018 (1.000, 1.037)	1.019 (0.997, 1.041)	1.016 (0.995, 1.038)
Stratified by Hyperlipidemia			
Yes	1.031 (1.016, 1.047)^***^	1.033 (1.017, 1.050)^***^	1.018 (0.999, 1.038)
No	1.050 (1.004, 1.098)^*^	1.051 (1.007, 1.096)^*^	1.054 (1.012, 1.097)^*^

a Model 1 adjusted for Age, Sex, and Races.

b Model 2 adjusted for Age, Sex, Races, Education, Smoke, and Alcohol.

c Model 3 adjusted for Age, Sex, Races, Education, Smoke, Alcohol, BMI, Hyperlipidemia, and Globulin.

d In the subgroup analysis by gender, the model was not adjusted for the stratification variable itself.

*p < 0.05, **p < 0.01, ***p < 0.001.

**Figure 3:**
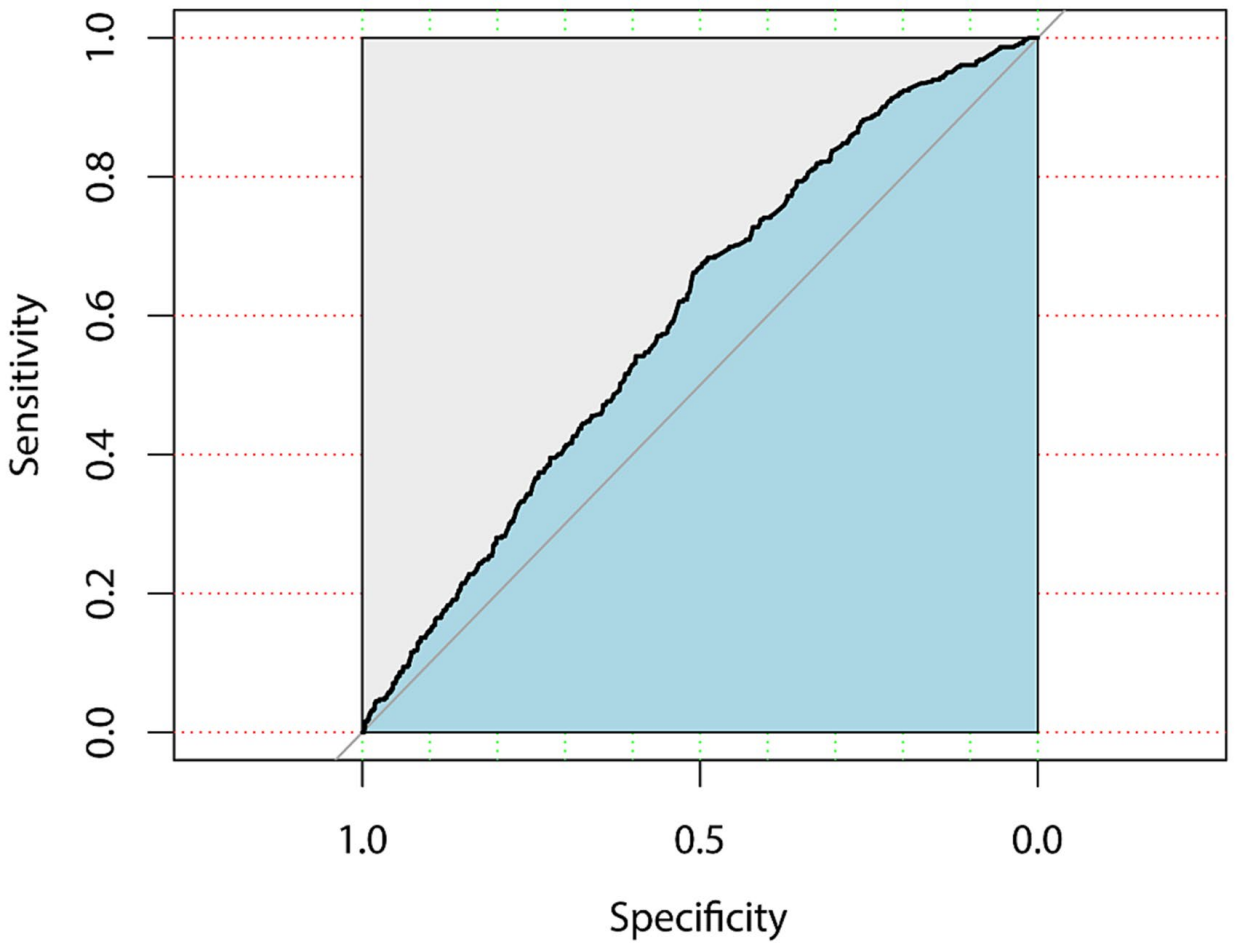
ROC curve for the frailty index in assessing the existence of significant fibrosis in NAFLD.

Within the subgroup analyses that were stratified according to BMI classifications, a positive correlation was identified between the FI and significant fibrosis among participants under/normal weight (OR = 1.077, 95% CI, 1.009–1.150) and overweight (OR = 1.040, 95% CI, 1.010–1.071) subjects in model3. Anyway, the positive association between the FI and significant fibrosis in NAFLD demonstrated variability in accordance with factors such as gender, BMI, and hyperlipidemia. The area under the Receiver Operating Characteristic (ROC) curve for the FI in assessing the existence of significant fibrosis in NAFLD was 0.612 (95% CI, 0.596–0.628), the ROC plot is presented in Figure 3.

## Discussion

With the progression of population aging, there is an escalating prevalence of physiological decline and age-associated frailty among the older populace. This diminishment curtails their capacity to effectively confront ailments or traumas, consequently engendering a heightened susceptibility to adverse consequences. In recent years, the relationship between hepatic fibrosis and frailty has increasingly captured the attention of the academic community (22). Through an in-depth analysis of NHANES, our findings suggest that significant hepatic fibrosis is an important risk factor for frailty in middle-aged and older adults. The correlation was more statistically significant for non-obese males without hyperlipidemia.

Existing evidence indicate that the deterioration of health functions significantly contributes to the onset of frailty. This underscores the significance of safeguarding health conditions as a key lifeline for maintaining overall well-being and vitality. While geriatric studies have traditionally concentrated on exploring the connection between specific disorders and disease outcomes, taking into account overall frailty can offer a more holistic understanding. This is because frailty serves as a common endpoint for various health dysfunctions (23). Utilizing frailty indices, such as the FI, defined as a heightened susceptibility to physiological stress stemming from functional decline in various organ systems, can be advantageous in examining this aspect. The FI can help predict the risk of mortality, guide treatment decisions, and provide a more comprehensive understanding of the patient’s health status (17). It is noteworthy that numerous studies have identified potential associations between the FI and a variety of age-related diseases, including heart failure (24), stroke (25), diabetes (26), and depression (27). The elucidated discoveries underscore the latent capability of evaluating comprehensive health condition as a means to comprehend the intricate nexus between factors of frailty and disease outcomes. Historical investigations have alluded to the fact that frailty is frequently observed in patients afflicted with NAFLD which is accompanied by advanced hepatic fibrosis or cirrhosis (28). This makes the evaluation of frailty assumes a pivotal role in managing patients with hepatic fibrosis.

Frailty and hepatic fibrosis are two interconnected conditions, and the connection between these two conditions lies in the impact of hepatic fibrosis on an individual’s physical strength and resilience (29). A systematic review found that as hepatic fibrosis advances, a deterioration in patients’ physical conditions is usually observed, triggering an increase in frailty (30). Factors such as fatigue, malnutrition and muscle wasting, commonly linked with advanced liver disease, are direct contributors to this increase in frailty. When NAFLD progresses to advanced stages, physiological resilience decreases and frailty ensues.

Several explanations can be offered to elucidate the potential mechanism of frailty in patients with significant fibrosis. First, in a large NAFLD cohort study performed by Koo et al. (31), sarcopenia was found to be significantly associated with significant fibrosis. At the pathophysiological echelon, alterations in the metabolic state of hepatic fibrosis engender a disequilibrium between energy requisites and intake, thereby instigating a metamorphosis in protein metabolism. This is particularly evident in the diminished circulating levels of branched-chain amino acids (bCAAs), which in turn accelerates muscular catabolism (32). The ratio of serum creatinine/serum cystatin C, as a surrogate marker for muscle mass, has been found to be significantly associated with frailty in multiple studies (33–35), consider that it is associated with falls, functional decline, disability and increased mortality in older adults.

Second, a study conducted by Leng et al. (36) revealed that pro-inflammatory markers such as IL-6 and tumor necrosis factor II were found to be elevated in individuals classified as frail. This highlights inflammation as a potential physiological source of frailty and suggests that it may serve as a biomarker for identifying high-risk patients. The intricate interplay among hepatocytes, macrophages, and hepatic stellate cells (HSCs), set within the context of the liver’s inflammatory and oxidative milieu, serves as a pivotal determinant in the pathogenesis of fibrosis (37, 38). Thus, it is undeniable that liver fibrosis is intrinsically linked to frailty through inflammatory responses and elevated levels of oxidative stress (39, 40). Moreover, in a large community-based cohort study, researchers have found that cognitive function may be poorer in high-risk patients with advanced fibrosis compared to low-risk patients, particularly in terms of executive function and abstract reasoning (41). Current explanations for the relationship between liver fibrosis and low cognitive function include oxidative stress, insulin resistance, and adipokine secretion (42). At the same time, among older adults, frailty is associated with poorer processing speed, sustained attention, working memory, and global cognition (43). From there, it is considered that significant hepatic fibrosis may be associated with frailty in the older people through altered cognitive status. The compounding effects of these biological processes underscore the confluence of systemic biological deterioration that typifies the frailty syndrome. This provides a plausible explanation for more than one interaction between significant hepatic fibrosis and frailty.

The relationship between significant hepatic fibrosis and the FI can have important clinical implications. For example, the coexistence of hepatic fibrosis and a high FI can stratify patients into higher risk categories, as both conditions can synergistically lower a patient’s physiological reserve and increase the risk of adverse outcomes. This stratification can be critical in managing patient care. Also, before considering a patient for a liver transplant or other major surgeries, the FI can be a valuable tool to assess their ability to withstand surgery and recover postoperatively. Those with higher frailty may require more rigorous preoperative optimization.

Interventions aimed at reducing frailty – such as nutritional support, physical activity, and muscle training – can also play a crucial role in managing hepatic fibrosis (31). It warrants acknowledgment that the interplay between frailty and hepatic fibrosis presents complexity, given their potential to reciprocally influence each other in numerous ways, thereby posing challenges to the efficacy of management strategies.

Yet, there is still a lack of comprehensive understanding regarding the influence of hepatic fibrosis on frailty (44) and the potential underlying mechanisms need more large-sample studies. It is also possible that hepatic fibrosis is associated with frailty through hepatocellular carcinoma as an ultimate consequence of liver disease and its impact on metabolic dysregulation and nutritional status. Exploring the possible link between hepatic fibrosis and frailty cannot be accomplished with cross-sectional data. To confirm the association between fibrosis and frailty, future studies must be longitudinal in design.

The research undertook an extensive examination of the correlation between the FI and substantial hepatic fibrosis using an expansive sample study in NAFLD. It also accounted variables that could skew the data, thereby enhancing the credibility of the findings. However, there are limitations to our studies. Firstly, the use of the FI and their respective scorings for frailty appraisal may have led to inaccuracies and unclear categorization. Secondly, the individuals of this research were all aged 50 years and above, so there may be limitations to the applicability of our findings to individuals below the age of 50. Finally, the liver stiffness measurement (LSM) threshold for evaluating hepatic fibrosis has shown variations across distinct studies, thus lacking a consensus standard for detecting steatosis.

## Conclusion

In summarization, an evaluation of frailty via the FI revealed a correlation with the significant hepatic fibrosis of NAFLD in middle-aged and older adults. Elevated FI exhibited a direct correlation with significant fibrosis in NAFLD patients, suggesting the FI may be a potential prospective biomarker for the assessment of hepatic fibrosis in this patient cohort. This association was particularly pronounced among male individuals, those categorized as non-obese, and subjects devoid of hyperlipidemia. Further studies, such as longitudinal studies, are needed to confirm the exact relationship between the FI and hepatic fibrosis and the underlying mechanisms.

## Data availability statement

The original contributions presented in the study are included in the article/Supplementary materials, further inquiries can be directed to the corresponding author.

## Ethics statement

The studies involving human data were conducted by utilizing the publicly available datasets provided by the National Health and Nutrition Examination Survey (NHANES), which is a program of studies designed to assess the health and nutritional status of adults and children in the United States and is managed by the Centers for Disease Control and Prevention (CDC). These datasets are collected by the NHANES with the appropriate consent and ethical approval from the participants, in compliance with the CDC’s ethical standards as stated on their website (https://www.cdc.gov/nchs/nhanes/). Given that our study did not involve direct interaction with human subjects and relied entirely on these de-identified, publicly available datasets, further ethical approval from our institution was not necessary.

## Author contributions

FA: Writing – original draft, Writing – review & editing. X-JL: Writing – review & editing. AA: Writing – review & editing. JL: Software, Writing – review & editing. YZ: Data curation, Writing – review & editing. YA: Methodology, Writing – review & editing. H-LG: Conceptualization, Writing – review & editing. Z-QZ: Conceptualization, Funding acquisition, Writing – review & editing.

## Footnotes

^1^ http://www.cdc.gov/nchs/nhanes.htm
