# Supplementary material for: Association between frailty and hepatic fibrosis in NAFLD among middle-aged and older adults: results from NHANES 2017–2020
Source: Front Public Health. 2024 Feb 8;12:1330221. doi: 10.3389/fpubh.2024.1330221 (PMC10883311; doi:10.3389/fpubh.2024.1330221)
Supplement: Supplementary file 1 [file Data_Sheet_1.docx]

**Supplementary Table 1.** Variables in the 49-Item frailty index and their respective scorings

| Variant |  |  |  |  |  | Scoring |  |  |  |  |  |  |  |  |  |  |
| --- | --- | --- | --- | --- | --- | --- | --- | --- | --- | --- | --- | --- | --- | --- | --- | --- |
| Cognition | |  |  |  |  |  |  |  |  |  |  |  |  |  |  |  |
| 1. Experience confusion/memory problems | | | | |  | Yes = 1, No = 0 | |  |  |  |  |  |  |  |  |  |
| Dependence | |  |  |  |  |  |  |  |  |  |  |  |  |  |  |  |
| 2. Managing money | | |  |  |  | Difficulty = 1, No Difficulty = 0 | | | |  |  |  |  |  |  |  |
| 3. Stooping, crouching, kneeling | | | |  |  | Difficulty = 1, No Difficulty = 0 | | | |  |  |  |  |  |  |  |
| 4. Lifting or carrying | | |  |  |  | Difficulty = 1, No Difficulty = 0 | | | |  |  |  |  |  |  |  |
| 5. House chore | |  |  |  |  | Difficulty = 1, No Difficulty = 0 | | | |  |  |  |  |  |  |  |
| 6. Preparing meals | | |  |  |  | Difficulty = 1, No Difficulty = 0 | | | |  |  |  |  |  |  |  |
| 7. Standing up from armless chair | | | |  |  | Difficulty = 1, No Difficulty = 0 | | | |  |  |  |  |  |  |  |
| 8. Getting in and out of bed difficulty | | | | |  | Difficulty = 1, No Difficulty = 0 | | | |  |  |  |  |  |  |  |
| 9. Using fork, knife, drinking from cup | | | | |  | Difficulty = 1, No Difficulty = 0 | | | |  |  |  |  |  |  |  |
| 10. Dressing yourself | | |  |  |  | Difficulty = 1, No Difficulty = 0 | | | |  |  |  |  |  |  |  |
| 11. Standing for long periods difficulty | | | | |  | Difficulty = 1, No Difficulty = 0 | | | |  |  |  |  |  |  |  |
| 12. Grasp/holding small objects | | | |  |  | Difficulty = 1, No Difficulty = 0 | | | |  |  |  |  |  |  |  |
| 13. Attending social event | | | |  |  | Difficulty = 1, No Difficulty = 0 | | | |  |  |  |  |  |  |  |
| 14. Push or pull large objects | | | |  |  | Difficulty = 1, No Difficulty = 0 | | | |  |  |  |  |  |  |  |
| 15. Walking for a quarter mile difficulty | | | | |  | Difficulty = 1, No Difficulty = 0 | | | |  |  |  |  |  |  |  |
| 16. Walking up 10 steps difficulty | | | | |  | Difficulty = 1, No Difficulty = 0 | | | |  |  |  |  |  |  |  |
| Depressive Symptoms | | |  |  |  |  |  |  |  |  |  |  |  |  |  |  |
| 17. Have little interest in doing things | | | | |  | Nearly every day = 1, More than half the days = 0.66, Several days = 0.33, Not at all = 0 | | | | | | | | | | |
| 18. Feeling down, depressed, or hopeless | | | | |  | Nearly every day = 1, More than half the days = 0.66, Several days = 0.33, Not at all = 0 | | | | | | | | | | |
| 19. Trouble sleeping or sleeping too much | | | | |  | Nearly every day = 1, More than half the days = 0.66, Several days = 0.33, Not at all = 0 | | | | | | | | | | |
| 20. Feeling tired or having little energy | | | | |  | Nearly every day = 1, More than half the days = 0.66, Several days = 0.33, Not at all =O | | | | | | | | | | |
| 21. Poor appetite or overeating | | | |  |  | Nearly every day = 1, More than half the days = 0.66, Several days = 0.33, Not at all = 0 | | | | | | | | | | |
| 22. Feeling bad about yourself | | | |  |  | Nearly every day = 1, More than half the days = 0.66, Several days = 0.33, Not at all = O | | | | | | | | | | |
| 23. Trouble concentrating on things | | | | |  | Nearly every day = 1, More than half the days = 0.66, Several days = 0.33, Not at all = 0 | | | | | | | | | | |
| Comorbidities | |  |  |  |  |  |  |  |  |  |  |  |  |  |  |  |
| 24. Arthritis | |  |  |  |  | Yes = 1, Suspect = 0.5 No = 0 | | | |  |  |  |  |  |  |  |
| 25. Thyroid problems | | |  |  |  | Yes = 1, Suspect = 0.5 No = 0 | | | |  |  |  |  |  |  |  |
| 26. Chronic bronchitis | | |  |  |  | Yes = 1, Suspect = 0.5 No = 0 | | | |  |  |  |  |  |  |  |
| 27. Cancer | |  |  |  |  | Yes = 1, Suspect = 0.5 No = 0 | | | |  |  |  |  |  |  |  |
| 28. Congestive heart failure | | | |  |  | Yes = 1, Suspect = 0.5 No = 0 | | | |  |  |  |  |  |  |  |
| 29. Coronary heart disease | | | |  |  | Yes = 1, Suspect = 0.5 No = 0 | | | |  |  |  |  |  |  |  |
| 30. Angina | |  |  |  |  | Yes = 1, Suspect = 0.5 No = 0 | | | |  |  |  |  |  |  |  |
| 31. Heart attack | |  |  |  |  | Yes = 1, Suspect = 0.5 No = 0 | | | |  |  |  |  |  |  |  |
| 32. Stroke | |  |  |  |  | Yes = 1, Suspect = 0.5 No = 0 | | | |  |  |  |  |  |  |  |
| 33. Blood pressure | | |  |  |  | Yes = 1, Suspect = 0.5 No = 0 | | | |  |  |  |  |  |  |  |
| 34. Diabetes | |  |  |  |  | Yes = 1, Suspect = 0.5 No = 0 | | | |  |  |  |  |  |  |  |
| 35. weak/failing kidneys | | |  |  |  | Yes = 1, Suspect = 0.5 No = 0 | | | |  |  |  |  |  |  |  |
| 36. Urinary Leakage | | |  |  |  | Yes = 1, Suspect = 0.5 No = 0 | | | |  |  |  |  |  |  |  |
| Hospital Utilization and Access to Care | | | | |  |  |  |  |  |  |  |  |  |  |  |  |
| 37. Self-rated health | | |  |  |  | Fair, poor = 1, Excellent, Very good, good = 0 | | | | | |  |  |  |  |  |
| 38. Health now compared with 1 year ago | | | | |  | Worse = 1, About the same, better = 0 | | | | |  |  |  |  |  |  |
| 39. Overnight hospital patient in past year | | | | | | Yes = 1, No = 0 | |  |  |  |  |  |  |  |  |  |
| 40. Frequency of health care use during past year | | | | | | None = 0, 1-5 = 0,5, More than 5 = 1 | | | | |  |  |  |  |  |  |
| 41. Number of prescribed medications | | | | |  | None = 0, 1-4 = 0.5, 5 and more = 1 | | | | |  |  |  |  |  |  |
| Physical Performance and Anthropometry | | | | |  |  |  |  |  |  |  |  |  |  |  |  |
| 42. Body mass index | | |  |  |  | <18.5, >30 = 1 | |  |  |  |  |  |  |  |  |  |
|  |  |  |  |  |  | 25-<30 = 0.5 | |  |  |  |  |  |  |  |  |  |
|  |  |  |  |  |  | 18.5-25 = 0 | |  |  |  |  |  |  |  |  |  |
| 43. Handgrip strength | | |  |  |  | MALE: |  |  |  | FEMALE: |  |  |  |  |  |  |
|  |  |  |  |  |  | For BMI ≤ 24, GS ≤ 29 | | |  | For BMI ≤ 23, GS ≤ 17 | | |  |  |  |  |
|  |  |  |  |  |  | For BMI 24.1-28, GS ≤ 30 | | |  | For BMI 23.1-26, GS ≤ 17.3 | | | |  |  |  |
|  |  |  |  |  |  | For BMI >28, GS ≤ 32 = 1 | | |  | For BMI 26.1-29, GS ≤ 18 | | |  |  |  |  |
|  |  |  |  |  |  |  |  |  |  | For BMI>29, GS ≤ 21 = 1 | | |  |  |  |  |
| Laboratory Values | | |  |  |  |  |  |  |  |  |  |  |  |  |  |  |
| 44. Glycohemoglobin (%) | | |  |  |  | 0%—5.7% = 0, >5.7% = 1 | | |  |  |  |  |  |  |  |  |
| 45. Red blood cell count (million cells/uL) | | | | | | M: 4.7-6.1 = 0, Other = 1 | | |  | F: 4.2-5.4 = 0, Other = 1 | | |  |  |  |  |
| 46. Hemoglobin (g/dL.) | | |  |  |  | M: 13.5-18 = 0, Other = 1 | | |  | F: 12-16 = 0, Other = 1 | | |  |  |  |  |
| 47. Red cell distribution width (%) | | | | |  | 11.6-14.6 = 0, Other = 1 | | |  |  |  |  |  |  |  |  |
| 48. Lymphocyte percent (%) | | | |  |  | 20-40 = 0, Other = 1 | | |  |  |  |  |  |  |  |  |
| 49. Segmented neutrophils percent (%) | | | | |  | 40-80 = 0, Other = 1 | | |  |  |  |  |  |  |  |  |
| **BMI, Body mass index; GS, grip strength.** | | | | |  |  |  |  |  |  |  |  |  |  |  |  |
